# Advanced directives’ knowledge among Portuguese palliative patients and caregivers: do the sociodemographic factors influence it? A cross-sectional survey

**DOI:** 10.1186/s12904-023-01203-7

**Published:** 2023-07-01

**Authors:** Catarina Sampaio Martins, Rui Nunes

**Affiliations:** 1 Palliative Medicine Service of Centro Hospitalar de Trás-os-Montes e Alto Douro, Avenida da Noruega, Lordelo, 5000-508 Vila Real, Portugal; 2 Faculty of Medicine, MEDCIDS-Department of Community Medicine, Information and Decision in Health, University of Porto, 4200-450 Porto, Portugal

**Keywords:** Palliative care, Advance directives, Proxy, Sociodemographic factors

## Abstract

**Background:**

The influence of demographic factors on the completion and knowledge of the Portuguese Advance Directives (PAD) and the Health Care Proxy’s (HCP) role is still not clear.

**Objectives:**

To identify sociodemographic features associated with knowledge and adherence to PAD and HCP in palliative care.

**Design:**

Cross-Sectional analysis of the sociodemographic data, PAD and HCP role knowledge, and PAD Register from a group of Portuguese palliative patients and their caregivers enrolled on the DAVPAL trial to test the PAD efficacy in promoting better concordance between patients and caregivers.

**Participants:**

One hundred twenty participants (60 palliative patients and 60 caregivers).

**Methods:**

After enrollment, the participants' sociodemographic data was acquired, their knowledge of PAD and the role of an HCP was questioned, and their prior register of the PAD was asked.

**Results:**

60 patients and 60 caregivers were included (*n*=120), with differences among them regarding age (*p*<.001), gender (*p*=.003), education (*p*<.001), job (*p*<.001), marital status (*p*=.043), and access to the internet (*p*=.003), but not regarding religion (*p*=.21). Only 13.3% of the participants were aware of PAD, 15.0% were aware of the HCP role, and 5.0% had previously filled a PAD. Religious beliefs (Non-Catholic) were the only sociodemographic factor significantly related to these three topics.

**Conclusions:**

There is low awareness of PAD and the HCP’s role in palliative care, and there is higher knowledge on these topics among non-Catholic individuals. End-of-life decisions seem to be influenced by similarities in religious beliefs between patients and HCP. Improving education is necessary, namely in palliative care.

**Trial registration:**

ClinicalTrials.gov ID NCT05090072. Retrospectively registered on 22/10/2021.

## Introduction

In all dimensions of health care, any treatment requires a patient’s informed consent, both regarding legal and ethical considerations. Therefore, the patient’s preferences of care after establishing a diagnosis are crucial criteria to fulfil this requirement [1]. Regarding health decisions, a high percentage of patients prefer active decisional control on how their treatments will be conducted throughout all the procedures [2]. Advance care planning (ACP), according to the consensus project commissioned by the European Association for Palliative Care, [3] is a process that “enables individuals to define goals and preferences for future medical treatment and care, to discuss these goals and preferences with family and health-care providers, and to record and review these preferences if appropriate.”

## Background

A population survey across Europe showed that although in Portugal, only 51% of the participants chose to die at home, a considerable percentage of European people prefer having control over their dying conditions and environment, namely by dying in the comfort of their homes [4, 5]. When patient’s directives are explicit, although more palliative care units are needed, hospitals register fewer unnecessary hospitalizations without affecting the patient’s satisfaction with the provided health care [6]. Also, when the patients’ advance directives are clear and objective allow better communication with their health care providers and their family members, with a significant impact on their quality of life since they are more secure that their health-related values and decisions will be assured once they are no longer able to express them [7].

In Portugal, ACP follows the legal Portuguese Advance Directives (PAD) in which patients can make a full description of their preferences in a document that functions as a living will and/or appoint a health care proxy (HCP), commonly a family member, which will assume the responsibility of enforcing and protecting the patients’ wishes when they are no longer able to express them [8–10]. Accordingly to the Portuguese law [10], the nominated HCP have the role and the power to decide about the healthcare that the patients will or not receive, on a substitutive judgement basis, when they are personally incapable of expressing their preferences autonomously.

Laranjeira *et al*. [11], who conducted an online survey on advanced care directives for improving the quality of care for patients at the end of life, including 1024 Portuguese adults, reported that a significant proportion (76.4%) have heard of ACP, but only 2.3% had ever filled in these types of directives.

The number of people that knows about the existence of PAD and that have actually registered the documents in the national database (which guarantees that their directives will be accessible in any hospital in the country), is still significantly low, [12] and this might be due to low efficiency in conveying information on ACP. In 2017, in the context of this low adherence to the ACP, the Portuguese Republic Assembly published a resolution that recommended the promotion of a campaign to publicize and encourage the registration of Living Wills, recognizing its importance and the need for improvement of the Portuguese population’s knowledge on this issue [13].

How information on ACP is conveyed was demonstrated to be of major significance, [14] and adequate communication towards ACP and end-of-life care initiatives results in a higher rate of completion of patient’s directives, as well as a better congruence between patient’s desires and treatments [15]. Assessing the quality of communication and the influence of different backgrounds and sociodemographic features on people’s knowledge of these directives and their actual registry is of the utmost importance. Ho et al. [16] evaluated how demographic backgrounds might affect ACP completion, also analyzing caregivers, showing that they influence ACP completion in terminally ill patients. In this study [16], they found that the patient’s age (older adults), clinical situation (non-oncologic), caregiver’s race (Caucasian) and income’s report denial were more related to the completion of the Advance Directives or the identification of an HCP. Inoue et al. [17] reinforce these findings, describing that increased age, female gender, Caucasian race and more favourable economic conditions positively influence ACP completion.

A study conducted in 2019 [18] also demonstrated that when patients talked about ACP before the directive’s registry, those conversations were more likely to be with family members or friends. Therefore, given the critical role that these people have in patient’s ACP considerations, it has been suggested that studies should focus not only on patients but also on HCP [19].

Su et al. [20] enhance the difficulties of the Health Care Proxy’s role and their lack of preparedness and emotional stress to accomplish their goals in the decision-making process, recommending the healthcare personnel’s attention and support on these subjects [20].

### Objectives

This study aims to identify sociodemographic features for both patients and HCP that influence the knowledge of PAD, the registration of health directives, and the knowledge of the role of an HCP.

We hypothesize that besides health professionals, all the people involved in health directives conversations and patient decisions, like family members and HCP, might influence the patient’s ACP. Investigating the sociodemographic characteristics of the population related to the completion of the ACP and their knowledge about the HCP role might allow a better understanding of the population’s adherence to this subject and improve the entire communication strategy and incentive to implement the ACP. In the same way, we consider that it is vital that family and caregivers understand the role of the nominated Health Care Proxy to guarantee that their decisions as surrogates are concordant with the patient’s wishes for the end-of-life period. Therefore, it is important to understand which and how the sociodemographic factors may be related to a better or worse knowledge of these concepts and to promote strategies that help in the population’s education.

## Methods

### Study design, patient selection and data acquisition

This study describes a cross-sectional analysis of the sociodemographic questionnaire applied to the participants of the DAVPAL study, which was a prospective, single-blinded, randomized controlled trial, accomplished in an inner land Portuguese Hospital, that enrolled patients in palliative care and their caregivers and was designed to assess the efficacy of the PAD when used as a communication tool between patients and caregivers on improving agreement and reliability between them, in end-of-life decisions (Catarina Sampaio Martins and colleagues. Do surrogates predict patient preferences more accurately after a physician-led discussion about advance directives? A randomized controlled trial. BMC Palliat Care 2022 Jul 12;21(1):122; registered on clinicaltrials.gov as NCT05090072).

The study was approved by the local Ethics Committee and the Institution´s Board.

The participants’ recruitment was made in their first consult on the Palliative Care Service, and the participation in the DAVPAL trial was voluntary, with no monetary incentives or others.

All the participants gave written consent to enrol on the trial, respecting all principles established both in the Declaration of Helsinki (World Medical Association) and in Europe´s Council Convention on Human Rights and Biomedicine. Also, all legal requirements on bioethics, data protection, and biomedical research were complied with. All the participants were assigned a code number, and all the data collected was anonymously registered under that code.

Patients were eligible if they fulfilled all the following criteria: adult male or females (18 or more years), intellectually, visually and auditorily capable, and cognitively unimpaired (confirmed with the Portuguese Validated Mini-Mental State Test [21, 22]). Patients were excluded if any of the following criteria were observed: a clinical condition that precluded the comprehension of the informed consent or any condition which would make the participant unsuitable for the study (e.g., non-compliance).

After enrolment in the DAVPAL trial, all 120 participants were asked to fill in a sociodemographic questionnaire and answer three dichotomous questions: if they had any previous knowledge of PAD, if they had ever filled in a PAD model before and if they had any previous knowledge on the role of an HCP. The sociodemographic questionnaire evaluated the following variables: gender, years and grade of education, current or previous job, working status, marital status, religious beliefs, and access to water, lighting, heating, television, mobile phone, and the internet. These questionnaires were self-administered to patients and caregivers in a designated room.

### Statistical analysis

A total sample size of 124 individuals was estimated using G*Power version 3.1.9.3, for a proportion difference of .20, a frequency of PAD knowledge of .10 in one of the groups, a type I error of .05 and a power of .80. All other statistical analysis was performed using IBM® SPSS® Statistics, version 27. Age was evaluated for normality using the Kolmogorov-Smirnov test for samples over 30 and Shapiro-Wilk otherwise, combined with the visual assessment of histograms and skewness and kurtosis measures. Since it was normally distributed in all settings, mean ± standard deviation was used for descriptive purposes. For qualitative variables, absolute and relative frequencies were used in the descriptive analysis.

For inferential analysis, a comparison of quantitative variables between two independent groups was performed using Student’s t-test. Association of sociodemographic variables with knowledge of PAD, on HCP and having previously filled a PAD model was assessed using the Chi-Square test when Cochran’s rule was met (less than 20% of expected values less than 5 and all equal or over 1); otherwise, categories were merged until meeting the previously mentioned assumptions or obtaining two categories per variable, in which case Fisher’s exact test was used, whenever more than one sociodemographic variable was associated with knowledge on PAD, on HCP, or on having previously filled a PAD model, a logistic model was used for multivariate inferential analysis.

A type I error of 0.05 was considered for all comparisons.

## Results

### Participants’ demographic data

A total of 120 participants were included, of which 60 were patients and 60 caregivers, meeting the accrual goals for sample size estimation, with an actual power of .78 for the abovementioned criteria. Demographics for each group are summarized in Table 1. Patients were significantly older than caregivers (70.6 ± 13.2 vs 58.6 ± 13.5, t(118) = 4.91, *p* < .001) and showed less years of education (χ^2^(2) = 24.4, *p* < .001) and a lower education grade (χ^2^(5) = 29.1, *p* < .001). Caregivers were mainly female (73.3%), while patients had a similar distribution regarding gender, with 46.7% female patients (χ^2^(1) = 8.89, *p* = .003). No differences between groups were seen regarding job description, but patients were more frequently retired than caregivers (78.3% vs 38.3%), with no patients actively working at the time of this study (χ^2^(2) = 40.0, *p* < .001). Caregivers were more frequently married (or in a similar marital status) than patients (80.0% vs. 63.3%, χ^2^(1) = 4.10, *p* = .043), while no differences were observed between groups considering religious belief (*p* = .21). No significant differences between patients and caregivers on access to water, lighting, heating, television, or mobile phones were observed; however, patients had lower access to the internet (40.0% vs 66.7%, χ^2^ (1) = 8.57, *p* = .003).

**Table 1:** Demographic characterization of patients and caregivers included in this study

	**Patients**	**Caregivers**	**Statistic**	***p*-value**	**Effect size**
**Age (years)**, mean ± SD	70.6 ± 13.2	58.6 ± 13.5	t(118) = 4.91	**< .001**	d = .90
**Gender**, n (%)					
Male	32 (53.3%)	16 (26.7%)	χ^2^(1) = 8.89	**.003**	Phi = .27
Female	28 (46.7%)	44 (73.3%)			
**Years of Education**, n (%)					
0-2 years	9 (15.0%)	2 (3.3%)	χ^2^(2) = 24.4	**< .001**	Cramer's V = .45
3-6 years	45 (75.0%)	28 (46.7%)			
≥ 7 years	6 (10.0%)	30 (50.0%)			
**Grade of Education**, n (%)					
Illiterate	7 (11.7%)	1 (1.7%)	χ^2^(5) = 29.1	**< .001**	Cramer's V = .49
Knows how to write and read	9 (15.0%)	4 (6.7%)			
Primary School	30 (50.0%)	15 (25.0%)			
Middle School	11 (18.3%)	17 (28.3%)			
High School	3 (5.0%)	12 (20.0%)			
University	0 (0.0%)	11 (18.3%)			
**Working status**, n (%)					
Social retirement	47 (78.3%)	23 (38.3%)	χ^2^(2) = 40.0	**< .001**	Cramer's V =.58
Sickness leave	10 (16.7%)	1 (1.7%)			
Unemployed	2 (3.3%)	4 (6.7%)			
Active worker	0 (0.0%)	30 (50.0%)			
Other	1 (1.7%)	2 (3.3%)			
**Marital status**, n (%)					
Single	9 (15.0%)	8 (13.3%)	χ^2^(1) = 4.10	.**043**	Phi = -.185
Married or similar	38 (63.3%)	48 (80.0%)			
Divorced	2 (3.3%)	0 (0.0%)			
Widower	11 (18.3%)	4 (6.7%)			
**Access to**, n (%)					
Water	59 (98.3%)	60 (100%)	#	n.s.	-
Lighting	60 (100%)	60 (100%)	N/A	N/A	N/A
Heating	52 (86.7%)	57 (95.0%)	χ^2^(1) = 2.50	n.s.	-
Television	60 (100%)	60 (100%)	N/A	N/A	N/A
Mobile Phones	59 (98.3%)	59 (98.3%)	#	n.s.	-
Internet	24 (40.0%)	40 (66.7%)	χ^2^(1) = 8.57	**.003**	Phi = .27
**PAD,** n (%)					
Knowledge on PAD	6 (10.0%)	10 (16.7%)	χ^2^(1) = 1.15	.28	Phi = .098
Ever filled a PAD model	1 (1.7%)	5 (8.3%)	#	.21	Phi = .15
Knowledge on HCP	6 (10.0%)	12 (20.0%)	χ^2^(1) = 2.35	.13	Phi = .14

Only significant differences and data related to PAD and HCP are reported in the table

*SD* Standard Deviation, *PAD* Portuguese Advance Directives, *n.s.* non-significant, *N/A* not applicable

### Knowledge on the Portuguese Advance Directives model

Only 16 participants (13.3%) had previous knowledge of the PAD model. Table 2 resumes the association between the previous knowledge of the PAD model according to the different demographic features assessed in this study. Participants that knew about PAD had significantly more years of education than those who did not (≥ 7 years: 56.3% vs. 26.0%, *p* = .020). An association between religious belief and knowledge about PAD was observed, with fewer catholic participants among those who knew about the PAD model (68.8% vs. 99.0%, *p* < .001). No other factors seemed to be associated with the knowledge of the PAD model.

**Table 2:** Demographic factors influencing knowledge on the Portuguese Advance Directives model

	**No knowledge on PAD model**	**Knowledge on PAD model**	**Statistic**	***p*-value**	**Effect size**
**Years of Education**, n (%)					
0-2 years	11 (10.6%)	0 (0.0%)	#	**.020**	Phi = .23
3-6 years	66 (63.5%)	7 (43.8%)			
≥ 7 years	27 (26.0%)	9 (56.3%)			
**Religion,** n (%)					
Catholic	103 (99.0%)	11 (68.8%)	#	**< .001**	Phi = .47
Jehovah’s Witness	0 (0.0%)	3 (18.8%)			
Agnostic	0 (0.0%)	1 (6.3%)			
Other	1 (1.0%)	1 (6.3%)			

Only significant differences are reported in the table

*PAD* Portuguese Advance Directives, *n.s.* non-significant, *N/A* not applicable

The impact of time of education and religion on the knowledge of PAD was assessed in multivariate analysis using a logistic regression model. An adjusted R^2^ of .273 was obtained, and religion maintained a significant impact on PAD model knowledge (B = 3.62, χ^2^_Wald_(1) = 9.70, *p* = .002).

### Filling a Portuguese Advance Directives (PAD) model

Six participants (5.0%) had previously filled in a PAD model, as seen in Table 3. The only factor for which an association was identified was religion, where participants who had previously filled in a PAD were mainly from religions other than Catholic, compared to those who had not filled in a PAD before (66.7% vs 1.8%, *p* < .001).

**Table 3:** Association between demographic factors and previous filling of a Portuguese Advance Directives model

	**Never filled a PAD**	**Previously filled a PAD**	**Statistic**	***p*-value**	**Effect size**
**Religion,** n (%)					
Catholic	112 (98.2%)	2 (33.3%)	#	**< .001**	Phi = .65
Jehovah’s Witness	0 (0.0%)	3 (50.0%)			
Agnostic	1 (0.9%)	0 (0.0%)			
Other	1 (0.9%)	1 (16.7%)			

Only significant differences are reported in the table

*PAD* Portuguese Advance Directives, *n.s.* non-significant, *N/A* not applicable

### Knowledge on Health Care Proxy (HCP) role

Among all participants, 18 (15.0%) knew the role of an HCP before entering this study. As described in Table 4, participants with no knowledge of the HPC role had a significantly lower education level (no schooling: 20.5% vs 0.0%, *p* = .040) and were mainly Catholic (98.0% vs 77.8%, *p* = .005) when compared to those who previously knew the role of an HCP. No other factors showed a significant association with the knowledge of the role of an HCP.

**Table 4:** Association between demographic factors and previous knowledge on a health care proxy role

	**No knowledge on HCP**	**Knowledge on HCP**	**Statistic**	***p*-value**	**Effect size**
**Grade of Education**, n (%)					
Illiterate	8 (7.8%)	0 (0.0%)	#	**.040**	Phi = .19
Knows how to write and read	13 (12.7%)	0 (0.0%)			
Primary School	38 (37.3%)	7 (38.9%)			
Middle School	25 (24.5%)	3 (16.7%)			
High School	11 (10.8%)	4 (22.2%)			
University	7 (6.9%)	4 (22.2%)			
**Religion,** n (%)					
Catholic	100 (98.0%)	14 (77.8%)	#	**.005**	Phi = .33
Jehovah’s Witness	0 (0.0%)	3 (16.7%)			
Agnostic	1 (1.0%)	0 (0.0%)			
Other	1 (1.0%)	1 (5.6%)			

Only significant differences are reported in the table

*HCP* Health Care Proxy, *n.s.* non-significant, *N/A* not applicable

The impact of education level and religion on the knowledge of the role of HCP was assessed in multivariate analysis using a logistic regression model. An adjusted R^2^ of .206 was obtained, and religion maintained a significant impact on DAV model knowledge (B = 2.42, χ^2^_Wald_(1) = 7. 40, *p* = .008).

## Discussion

Advance directives on health care in Portugal is a relatively recent topic, with the first legislative documents published and approved in July 2012 [10]. One of the main goals of this study was to assess whether patients and caregivers in the context of palliative care were aware of the existence of PAD. A low level of knowledge of these directives was observed since only 13.3% of the participants knew about PAD, and only 5.0% had previously filled in a PAD. These results somewhat conflict with the publication from Laranjeira *et al*. [11], where a significant proportion (76.4%) reported having heard of ACP, although only 2.3% had ever filled in these types of directives. These differences might be due to the fact that the participants in that study were younger (40.3 ± 11.4 years old) and had a higher proportion of university (or similar) enrolment (79.9%) compared to our sample. Also, the majority were healthcare professionals (71.1%), which might influence their knowledge of ACP, namely PAD. On the other hand, our results are consistent with those reported by other authors on other populations, namely on Hong Kong adults, where only 14.3% reported knowledge of ACP, [23] and on Chinese-American elders (21% reported previous knowledge of ACP, with 10% having previously filled in one directive) [24]. It should be noticed that ACP is known to be influenced by cultural differences, [25] which might relate to different results reported in other populations, such as those in Korean-American elders, with 35% reporting knowing about ACP [26].

Our results might reflect the lack of information available to the general population on this topic, as well as the inadequate importance assigned to these subjects, which should be aspects to address in programs dedicated to improving the Portuguese overall population health literacy, as proposed by some authors [8].

Our study encompassed patients in palliative care and their caregivers. As expected, significant differences were observed between these groups regarding age, gender, level and time of education, working and marital status, and access to the Internet. Although some of these factors have been reported as influencing decisional control preferences, such as higher education, [2] we opted to analyze participants altogether since both these groups are involved in end-of-life decisions. Differently from previous studies [16, 17] our results did not find significant associations between the participants’ age or gender and the knowledge or register of the ACP, or the knowledge of the HCP role, probably due to the small number of participants that were aware of these subjects.

In univariate analysis, longer time of education (a possible surrogate measure of education level) showed a significant association with higher rates of knowledge of PAD, consistent with these patients’ preferences for having control over their decisions, as mentioned above [2]. These results might also reflect insufficient health policies and serious flaws in the ACP divulgation and promotion to reach all population clusters, including less literate people.

In our sample of participants, we noticed that only 40% of the patients and 66.7% of the caregivers had access to the Internet, which might constitute a barrier to the ACP process. The literature also describes numerous web-based tools to support ACP adherence and registration worldwide [27]. However, we found no influence of the internet availability in the knowledge and register of the ACP, nor the knowledge of the HCP, which reinforces the idea that the population awareness on these subjects is insufficient.

Religion stood out as the recurring sociodemographic factor influencing the knowledge of PAD, on the role of an HCP, and on having previously filled in a PAD, both in univariate and multivariate analysis. In fact, non-Catholic participants tended to show better knowledge of these topics and better ACP completion rates. Studies addressing the influence of religion on ACP completion are somewhat conflicting, with no determinant association found in a systematic review [28]. Other publications showed a significant association of the similarity of religious values with the comfort that patients felt with their HCP, as they were more confident that the decisions made by the HCP reflected identical values [29].

In Southern Europe, religion was also found to have an influence on specific end-of-life decisions, such as those regarding pain relief treatments and end-of-life directives, with Catholics more prone to pro-life decisions in Portugal, Spain, and Italy [30]. Trarieux-Signol *et al.* [31] reported an association between the will to meet a religious representative and writing an ACP [31]. Although these authors also found a significantly higher rate of HCP assignment in married patients, no association of marital status with knowledge of the role of an HCP was observed in our study [31].

Our results might also be explained by the fact that some religious beliefs might directly influence the decision on some medical procedures, such as blood transfusions for Jehovah's Witness patients, underlining the need for these patients to seek more information on ACP and on the designation of an HCP, to ensure that their religious beliefs are respected by the health care practitioners.

In this particular case, literature shows that in some circumstances, many healthcare practitioners would give a Jehovah's Witness a blood transfusion, even knowing that it would be against the patient’s wishes [32].

These facts might justify the association of religious beliefs with the ACP process, as these social groups probably search for legal instruments to help them accomplish their wishes, even when ethical constraints might emerge.

### Limitations

This study included a small group of participants (60 patients and 60 caregivers) from a rural land of the Portuguese territory, precluding the inference of the results to the general population. Also, our group of participants had inequalities regarding gender distribution (60% were women), had no racial diversity (all the participants were Caucasian), low religious diversity (more than 90% were catholic) and globally had low levels of education (95% of the patients and 61,7% of the caregivers attended less than middle school), which might have influenced the results. We cannot exclude that some participants had difficulties understanding and filling in the sociodemographic questionnaire.

However, the positive results obtained on the influence of religious beliefs on ACP knowledge and completion must not be underestimated.

### Implications

Future studies are needed to address the ACP process and the sociodemographic factors that might influence its knowledge and adherence so that health policies and clinical practitioners identify targets to improve the population literacy on these subjects. The importance of the ACP process must be enlightened, and the role of the HCP must be cleared, to ensure that the patient’s autonomy is respected until the end of their lives. The healthcare practitioners, along with the governmental policies, are crucial in the promotion of the ACP, and in the explanation of its content to the general population and patients.

In the palliative care context, this assumes greater importance as the patient’s clinical conditions progressively deteriorate, and their decision-making capacity is lost.

Concerning our study’s results, as the majority of the Portuguese population is Catholic (about 81%) [33], there is a need for a higher sample size in order to establish the influence of specific religious beliefs on these topics. Also, a more heterogeneous sample, regarding age, gender or education levels, must be enrolled to allow inferences to the general population.

## Conclusions

In summary, the present study was designed to assess the knowledge of patients in palliative care and their caregivers on PAD and the role of HCP. We found that a low proportion of patients and caregivers were aware of PAD and the role of an HCP. We additionally observed that non-Catholic participants have higher consciousness on these topics. Further studies are warranted to demonstrate the influence of specific religious beliefs on ACP awareness and completion. There is a need for improvement regarding the access to information on ACP and HCP role to the Portuguese population to ensure that patients in palliative care have access to better health care.

## Data Availability

The datasets used and/or analyzed during the current study are available from the corresponding author on reasonable request.

## Abbreviations

ACP: Advance Care Planning
PAD: Portuguese Advance Directives
HCP: Health Care Proxy
